# Supplementary material for: Mitochondrial dynamics resolve physical-social tensions and challenges through adaptable multi-objective optimisation
Source: bioRxiv. 2026 Sep 22:2026.09.22.753427. Preprint. [Version 1] doi: 10.64898/2026.09.22.753427 (PMC13622113; doi:10.64898/2026.09.22.753427)

bioRxiv preprint doi: https://doi.org/10.64898/2026.09.22.753427; this version posted September 23, 2026. The copyright holder for this preprint (which was not certified by peer review) is the author/funder, who has granted bioRxiv a license to display the preprint in perpetuity. It is made available under a CC-BY 4.0 International license.

Supplementary Information

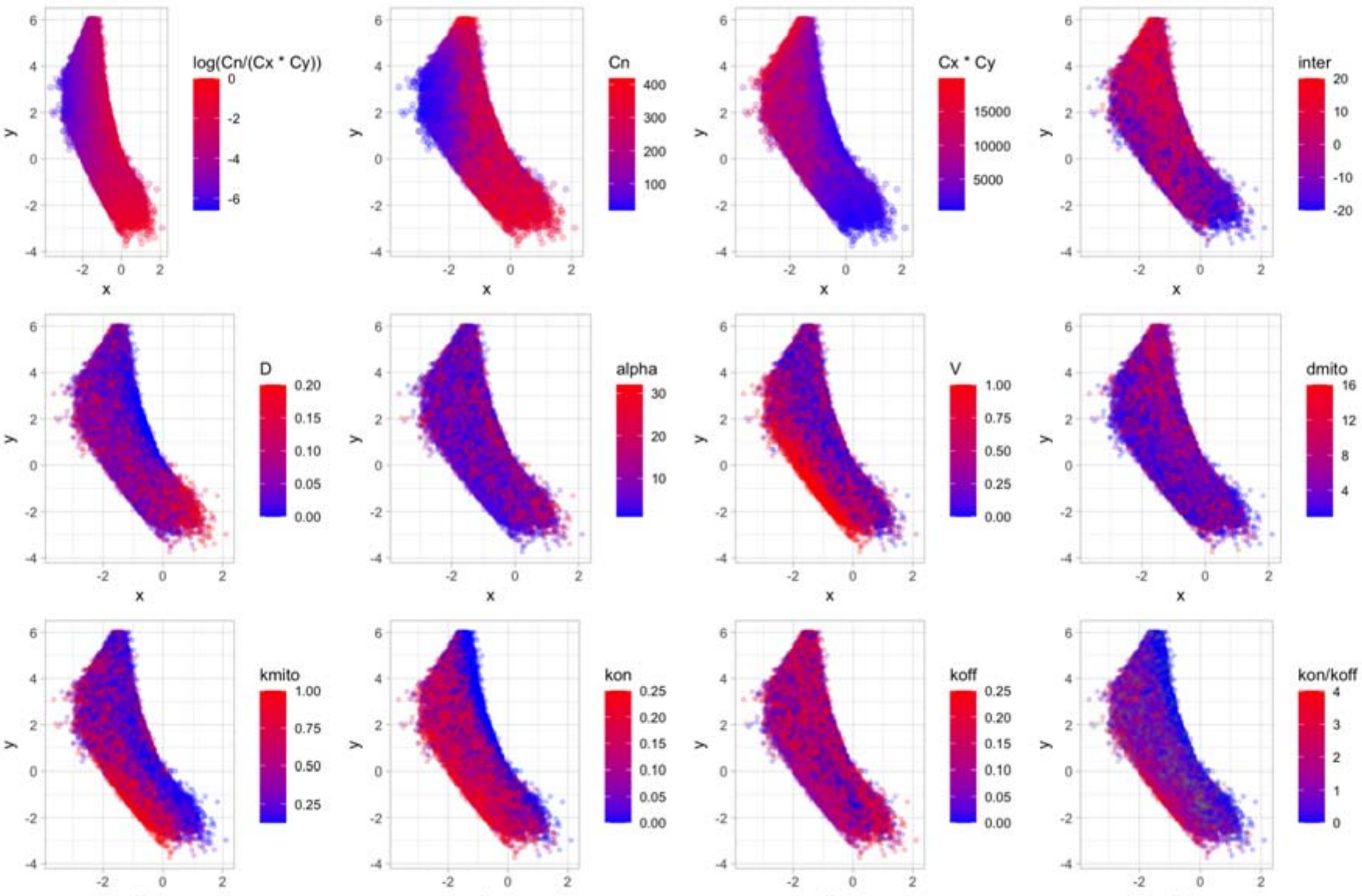

Supplementary Figure 1. **Physical-social morphospace with different simulation parameters.** In each plot, every point is a simulation output in the clumping-loneliness morphospace from Fig. 3. The value of the given physical parameter for each simulation is plotted as the colour scale. Mitochondrial density, for example, in part determines the position in the clumping-loneliness tradeoff. Higher ballistic velocity V tends to drive the system towards the optimal Pareto front.

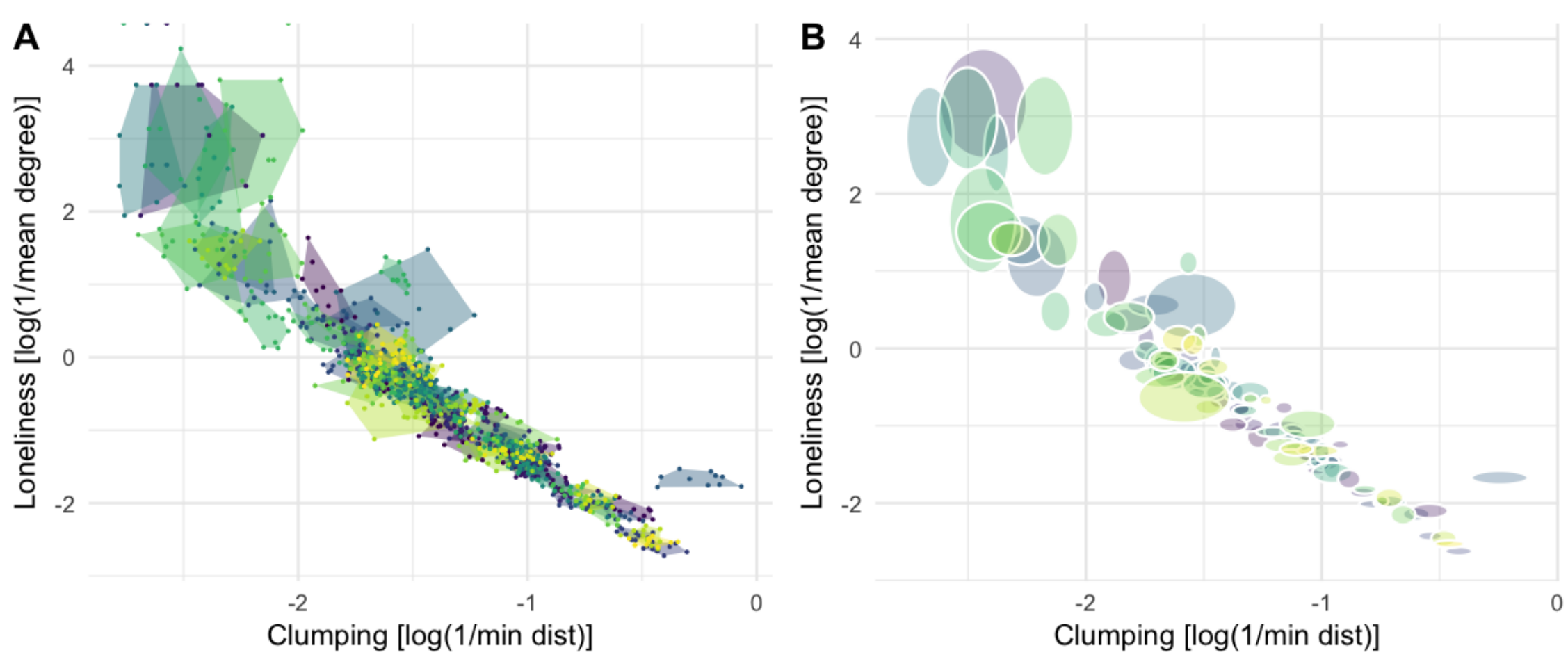

Supplementary Figure 2. **Set-valued optimisation.** As each simulation is stochastic (for example, with random initial conditions and diffusive steps), each parameterisation of the model generates a set of points in physical-social morphospace. Here two visualisations of this set-valued behaviour are given for 10 samples from each of a subset of 100 parameterisations: **(A)** convex hulls of the set arising from each parameterisation, and **(B)** ellipses centred on the mean and with axes giving the standard error on the mean behaviour.

bioRxiv preprint doi: https://doi.org/10.64898/2026.09.22.753427; this version posted September 23, 2026. The copyright holder for this preprint (which was not certified by peer review) is the author/funder, who has granted bioRxiv a license to display the preprint in perpetuity. It is made available under a CC-BY 4.0 International license.

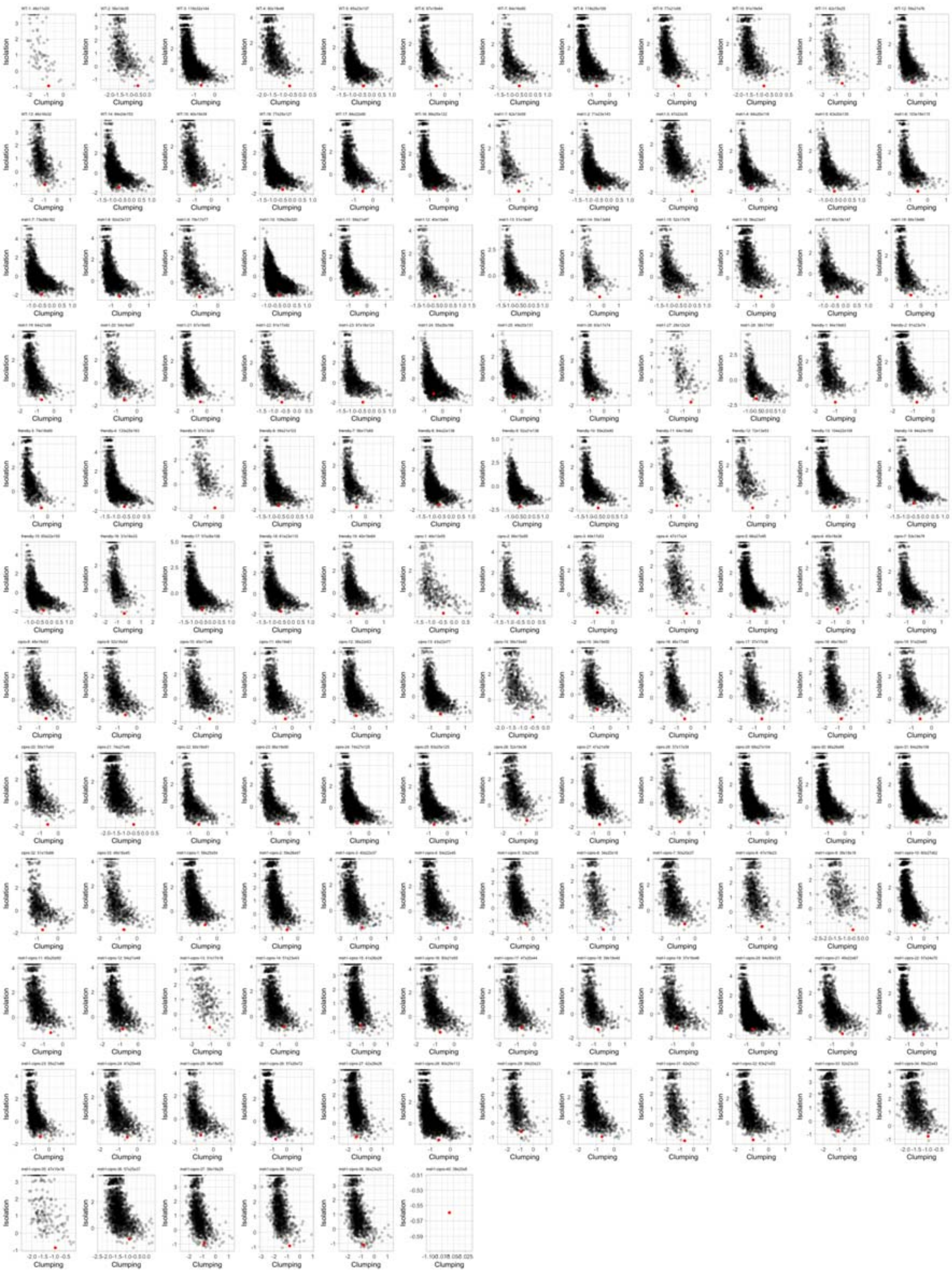

Supplementary Figure 3. **Cell-geometry-specific morphospaces.** Each panel corresponds to one single-cell experimental observation. The subset of simulation parameterisations matching that cell's geometry (cell dimensions within 10um, mitochondrial count within 10) are visualised in the physical-social morphospace (grey) and the experiment's position in morphospace is given in red.

bioRxiv preprint doi: https://doi.org/10.64898/2026.09.22.753427; this version posted September 23, 2026. The copyright holder for this preprint (which was not certified by peer review) is the author/funder, who has granted bioRxiv a license to display the preprint in perpetuity. It is made available under a CC-BY 4.0 International license.

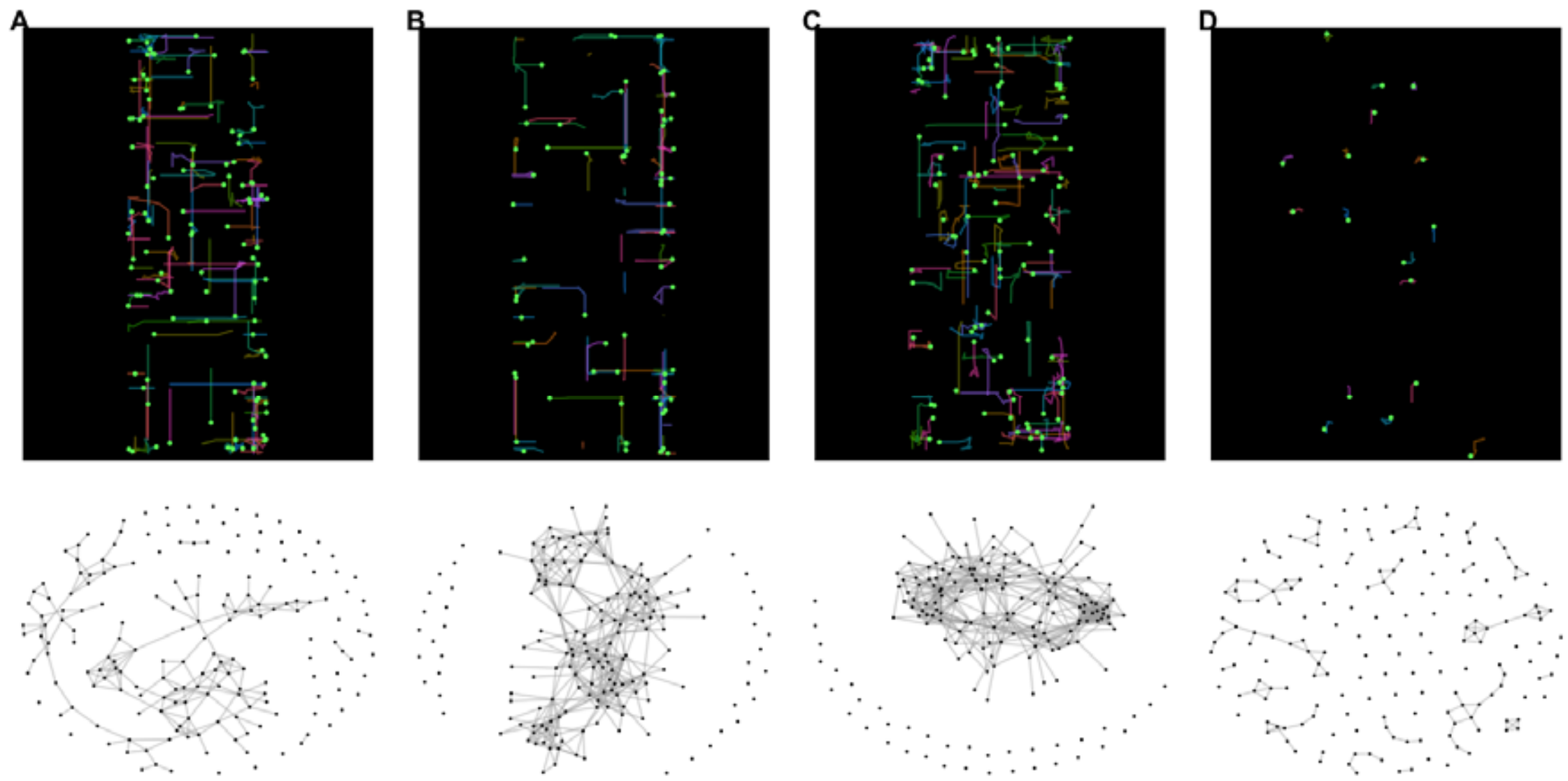

Supplementary Figure 4. **Illustration of simulations on the Pareto front. (A-B)** Two simulations from parameterisations on the Pareto front (Fig. 3A) near the region occupied by biological observations. **(C)** Simulation from parameterisation on the Pareto front in the highly social, but clumped regime. **(D)** Simulation from parameterisation on the Pareto front in the highly-spread, but isolated regime.

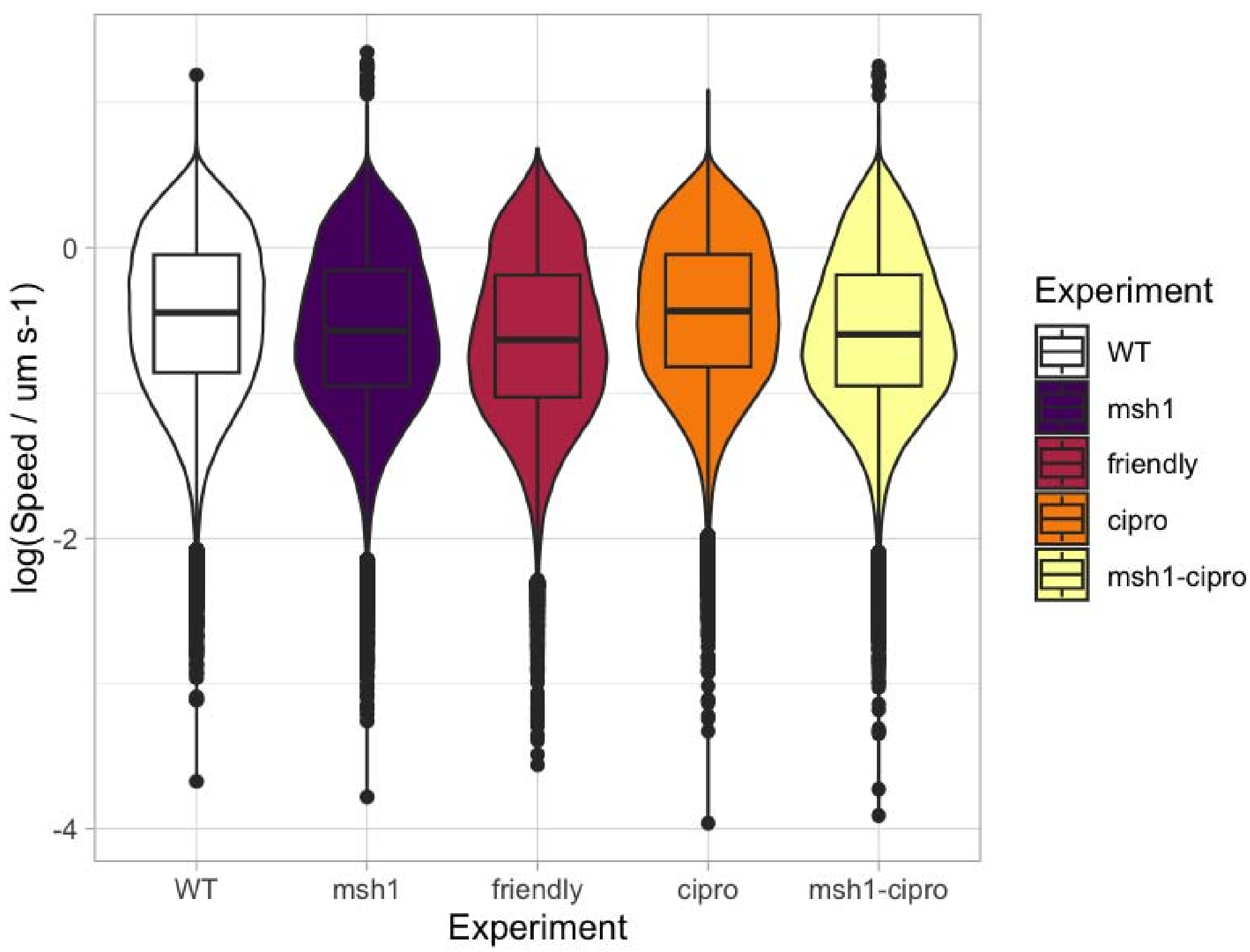

Supplementary Figure 5. **Distributions of mitochondrial speeds across the different experiments.**

bioRxiv preprint doi: https://doi.org/10.64898/2026.09.22.753427; this version posted September 23, 2026. The copyright holder for this preprint (which was not certified by peer review) is the author/funder, who has granted bioRxiv a license to display the preprint in perpetuity. It is made available under a CC-BY 4.0 International license.

Supplementary Video 1. **Example videos of observed mitochondrial dynamics and social network construction.** Videos of the raw microscopy imaging, the segmented and tracked particles from video analysis, and the consequent social network evolving over time. From top to bottom: two mtGFP cells (modelling wildtype), *msh1* mutant, *msh1-cipro* treatment.

https://github.com/StochasticBiology/optimal-mitos/blob/main/summary-videos/several-experiments.mp4

Snapshot:

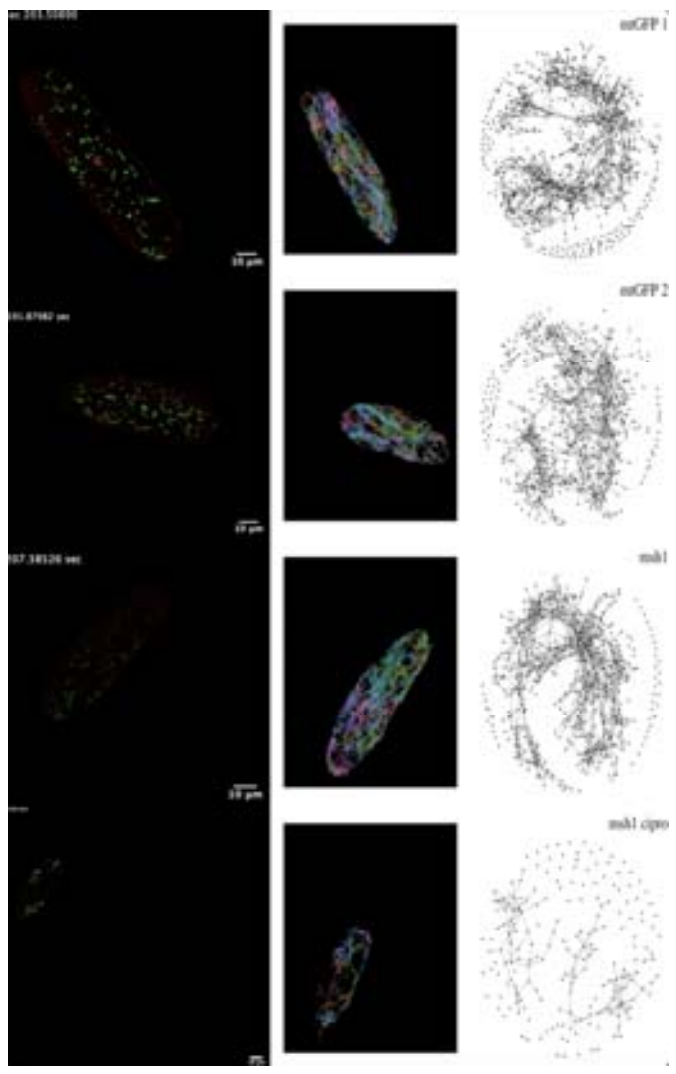

Supplementary Video 2. **Example video of simulated mitochondrial dynamics and social network construction.** (top) A parameterisation with a combination of cytoskeletal motion and diffusion, giving moderate spread and social connectivity. (centre) A parameterisation favouring physical spread over social connectivity. (bottom) A parameterisation favouring social connectivity over physical spread.

https://github.com/StochasticBiology/optimal-mitos/blob/main/summary-videos/several-simulations.mp4

Snapshot:

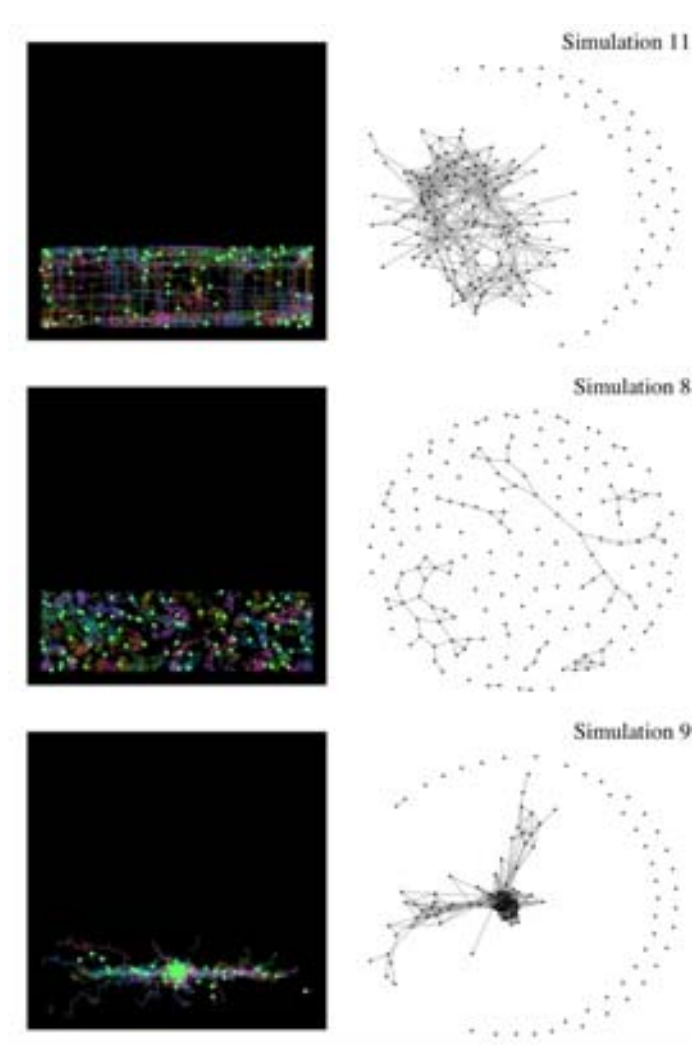

Supplement: Supplementary file 1 [file NIHPP2026.09.22.753427v1-supplement-3.pdf]
